# The role of cGMP-signalling and calcium-signalling in photoreceptor cell death: perspectives for therapy development

**DOI:** 10.1007/s00424-021-02556-9

**Published:** 2021-04-16

**Authors:** Soumyaparna Das, Yiyi Chen, Jie Yan, Gustav Christensen, Soumaya Belhadj, Arianna Tolone, François Paquet-Durand

**Affiliations:** Institute for Ophthalmic Research, University of Tübingen, Elfriede-Aulhorn-Strasse 7, 72076 Tübingen, Germany

**Keywords:** Retina, Ca^2+^, Protein kinase G, PKG, CNG channel, cGMP, Photoreceptor degeneration

## Abstract

The second messengers, cGMP and Ca^2+^, have both been implicated in retinal degeneration; however, it is still unclear which of the two is most relevant for photoreceptor cell death. This problem is exacerbated by the close connections and crosstalk between cGMP-signalling and calcium (Ca^2+^)-signalling in photoreceptors. In this review, we summarize key aspects of cGMP-signalling and Ca^2+^-signalling relevant for hereditary photoreceptor degeneration. The topics covered include cGMP-signalling targets, the role of Ca^2+^ permeable channels, relation to energy metabolism, calpain-type proteases, and how the related metabolic processes may trigger and execute photoreceptor cell death. A focus is then put on cGMP-dependent mechanisms and how exceedingly high photoreceptor cGMP levels set in motion cascades of Ca^2+^-dependent and independent processes that eventually bring about photoreceptor cell death. Finally, an outlook is given into mutation-independent therapeutic approaches that exploit specific features of cGMP-signalling. Such approaches might be combined with suitable drug delivery systems for translation into clinical applications.

## Introduction

The retina is a neuronal tissue devoted to the conversion of light-stimuli into electrochemical signals that can be interpreted by the central nervous system. The key conversion step is performed by photoreceptors, which are compartmentalized cells with an outer segment (OS) harboring the components of the phototransduction cascade, an inner segment (IS) containing mitochondria, a cell body with nucleus and organelles, and a synaptic region providing for connectivity with second-order neurons. Two main types of photoreceptors can be distinguished: rod photoreceptors (rods) and cone photoreceptors (cones). Rods respond to dim light and enable vision at night, whereas cones respond to bright daylight. In humans, cones are essential for high-resolution color vision [46].

**Fig. 1:**
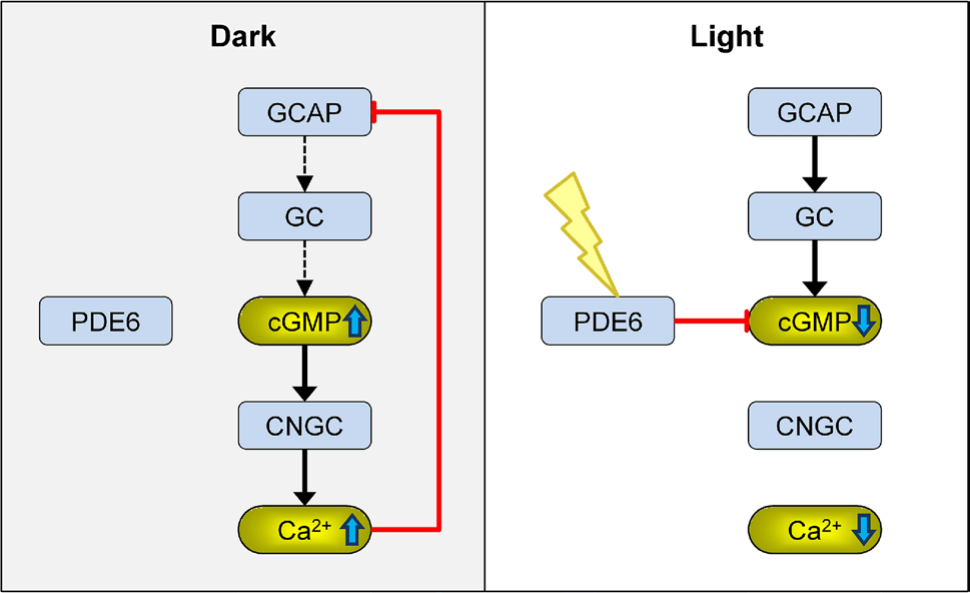
Interplay of cGMP and Ca^2+^ in photoreceptor outer segments. In darkness, Ca^2+^ prevents guanylyl cyclase–activating protein (GCAP) from activating retinal guanylyl cyclase (GC). GC produces cGMP, which opens the cyclic nucleotide–gated channel (CNGC), allowing for influx of Ca^2+^. In light, phototransduction leads to activation of phosphodiesterase-6 (PDE6), which hydrolyses cGMP, closing CNGC and stopping Ca^2+^ influx. This in turn leads to disinhibition of cGMP synthesis. Mutations in genes encoding for any of these proteins lead to dysregulation of cGMP and Ca^2+^ homoeostasis and can cause retinal degeneration (RD)

The phototransduction cascade relies to a large extent on the interplay of two essential signalling molecules: cyclic-Guanosine-Mono-Phosphate (cGMP) and Ca^2+^. In the dark, high levels of cGMP in photoreceptor OSs activate the cyclic nucleotide–gated channel (CNGC) to allow for an influx of Na^2+^ (≈80% of ion flux) and Ca^2+^ ions (≈20%) [48]. cGMP is synthesized by retinal guanylyl cyclase (GC) [45]. High levels of Ca^2+^ inhibit guanylyl cyclase–activating protein (GCAP), restricting GC activity [101], and providing for a negative feedback loop that limits photoreceptor cGMP to its physiological range of 1–5 µM [10, 17, 34, 79]. In light, the activation of phosphodiesterase-6 (PDE6) leads to cGMP hydrolysis and closure of CNGC. In turn, this leads to the disinhibition of GC and cGMP synthesis, allowing for a rapid restoration of cGMP levels once darkness sets back in [64] (Fig. 1).

**Fig. 2:**
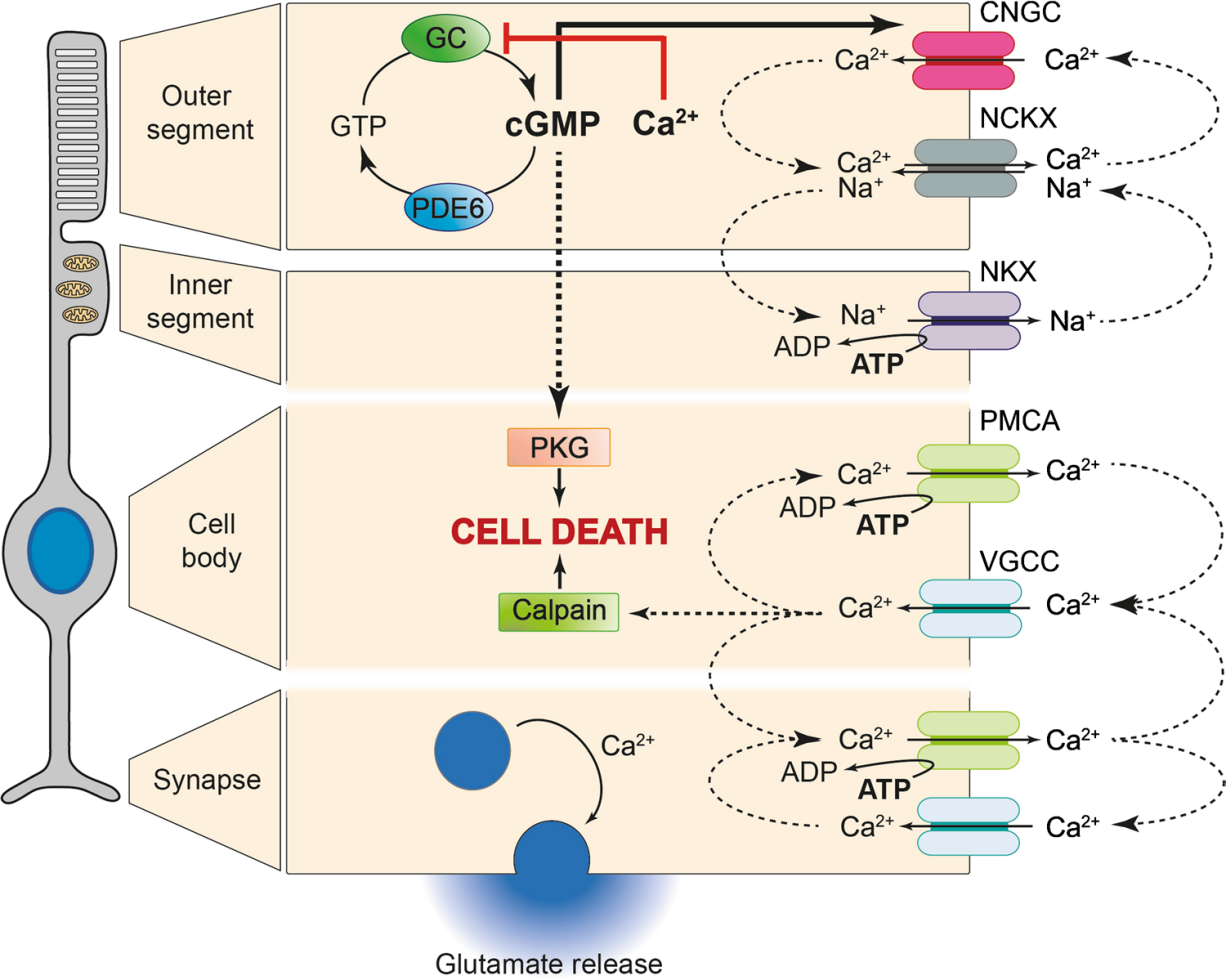
cGMP and Ca^2+^ signalling in different photoreceptor compartments. In RD-type diseases, photoreceptor degeneration was connected to high levels of cGMP and Ca^2+^. cGMP activates protein kinase G (PKG), which is linked to cell death, and cyclic nucleotide–gated ion channel (CNGC), promoting Ca^2+^ influx in the outer segment. In turn, Ca^2+^ can inhibit guanylate cyclase (GC), which converts GTP to cGMP. The cGMP signal is normally terminated by phosphodiesterase 6 (PDE6). Several channels are responsible for Ca^2+^ homeostasis: The Na^+^/Ca^2+^/K^+^ exchanger (NCKX) promotes Ca^2+^ efflux for Na^+^ influx. Excess Na^+^ is then expelled by the ATP-driven Na^+^/K^+^ exchanger (NKX) in the inner segment. Plasma membrane Ca^2+^ ATPase (PMCA) also extrudes Ca^2+^ in exchange for ATP hydrolysis. In the synapse and cell body, voltage-gated calcium channel (VGCC) is responsible for Ca^2+^ influx, which may activate calpain-type proteases to precipitate cell death. In the synapse, Ca^2+^ stimulates glutamate-containing vesicles to fuse with the membrane, regulating glutamate release

The CNGC-mediated influx of Na^+^ and Ca^2+^ in the dark needs to be compensated for by other ion channels and transporters. Ca^2+^ ions are exported from the OS by the Na^+^/Ca^2+^/K^+^ exchanger (NCKX), which uses high extra-cellular to intra-cellular electrochemical gradients of Na^+^ and K^+^ ions to drive out Ca^2+^ [22, 59, 83, 87]. The Na^+^ ions flowing into the OS through both CNGC and NCKX diffuse to the IS where they are exported by the ATP-driven Na^+^/K^+^ exchanger (NKX) (Fig. 2) [110]. The continuous influx of Na^+^ ions in the OS and corresponding outflux of K^+^ constitutes the so-called dark current, which ultimately is driven by the ATP-dependent NKX [63]. Light stimulation leads to hydrolysis of cGMP resulting in the deactivation of CNGCs and abatement of the dark current. Because Ca^2+^ extrusion through NCKX continues, the cytoplasmic Ca^2+^ decreases. This light-induced drop of intracellular Ca^2+^ constitutes a major signal for recovery and adaptation following light exposure [33, 80, 115].

Hereditary retinal degenerations (RDs) affect retinal photoreceptors, and in most cases these diseases are untreatable [43, 77]. Within the group of RD-type diseases, adult-onset retinitis pigmentosa (RP) is the most common, with a prevalence of approximately 1:3500 [8]. In RP, the genetic defect can reside in rod or RPE specific genes, leading to a primary rod degeneration, which then entails a secondary cone degeneration. Another RD-type disease is achromatopsia where the genetic defect resides in cone-specific genes bringing about a primary cone degeneration, usually without affecting rod photoreceptor viability [58]. While the mechanisms governing photoreceptor cell death in RD are still incompletely understood, for the past 25 years, research into these mechanisms has focused on apoptosis and a supposedly detrimental role of excessive intracellular Ca^2+^-levels [26, 65]. More recently, the focus has shifted towards non-apoptotic cell death mechanisms that are Ca^2+^-independent and in which cGMP-signalling plays a preeminent role [3, 77]. Photoreceptor cGMP accumulation has been observed in a number of murine models, for instance, in mice suffering from loss-of-function mutations in the *Aipl1*, *Cngb1*, *Pde6b*, *Prph2*, and *Rho* genes [3, 69, 81]. It appears likely that elevated photoreceptor cGMP levels are present in a number of yet other RD-gene mutations [71]. Overall, this highlights a possible general pathway, common to different disease genes and mutations, that could be targeted for the development of a broadly applicable therapeutic intervention for RP.

In this review, we summarize key aspects of cGMP-signalling and Ca^2+^-signalling relevant for hereditary photoreceptor degeneration. Indeed, it appears that both too low and too high cGMP and/or Ca^2+^ signalling may be lethal for photoreceptors, when present during extended periods of time [21]). We lay out the relevance of cGMP and Ca^2+^ signalling for phototransduction and normal photoreceptor physiology. We then detail the potential roles that Ca^2+^ permeable channels and downstream Ca^2+^-dependent processes may play in degenerative events. Eventually, we highlight recent evidence indicating how exceedingly high photoreceptor cGMP levels and cGMP-dependent mechanisms set in motion cascades of Ca^2+^-dependent and Ca^2+^-independent processes that bring about photoreceptor cell death.

## Crosstalk between cGMP and Ca^2+^-signalling

### CNG channels: activation and Ca^2+^ influx

CNGCs are heterotetrameric ion channels expressed in the OS membrane of rods and cones, where they mediate Na^+^ and Ca^2+^ influx. Rod photoreceptor CNGC is composed of CNGA1 and CNGB1a subunits expressed in a 3:1 ratio [42]. Cone photoreceptors, on the other hand, may express CNGA3 and CNGB3 subunits in a 1:1 ratio [74]. In both rods and cones, CNGCs are opened by cGMP binding, and they constitute one of the major sources for Ca^2+^ influx in photoreceptors. Because Ca^2+^ has been suggested as a major disease driver for photoreceptor degeneration [26], CNGC may constitute an attractive target for therapeutic interventions [68].

As shown in Fig. 1, Ca^2+^ influx in photoreceptor OS mediates inhibition of cGMP synthesis in a regulatory feedback loop that normally ensures that both cGMP and Ca^2+^ levels in the OS are kept within physiological ranges. However, when this feedback loop is broken by mutations in RD-genes, the result can be pathological changes in the intracellular concentrations of either cGMP or Ca^2+^ or both at once. For instance, loss-of-function mutations in *PDE6* genes cause an elevation of photoreceptor cGMP levels, overactivate CNGC, increase Ca^2+^ influx, and cause photoreceptor cell death [23, 57]. Conversely, loss-of-function mutations in CNGC genes could result in Ca^2+^ depletion, disinhibition of GC, exceedingly high cGMP production, and again photoreceptor cell death [3, 38, 112]. Hence, these two types of RD mutations might represent opposing ends when it comes to photoreceptor Ca^2+^ levels, high in case of PDE6 mutations and low in CNGC mutations. Both types of genetic defects invariably result in photoreceptor death, with the common point being high photoreceptor cGMP. On the other hand, it is important to consider that Ca^2+^ channels and exchangers are tightly coupled in a cellular system, therefore reduced function of CNGC may be compensated in a cell by decreased activity of exchangers. At any rate, high levels of cGMP will overactivate cGMP-dependent protein kinase G (PKG) [108, 112], the activity of which was found to be both necessary and sufficient to mediate cGMP-dependent photoreceptor cell death [69].

### PKG and its role in photoreceptor cell death

The prototypic cellular target for cGMP-signalling is PKG [27]. PKG is a serine/threonine-specific protein kinase, and in mammals, there are two different genes coding for three different enzyme isoforms: The *PRKG1* gene gives rise to the alternatively spliced α and β isoforms of PKG1, while the *PRKG2* gene encodes for PKG2 [37]. To be activated, PKG requires conformational changes to liberate the catalytic site. These changes are induced by binding of cGMP to the regulatory domain, which blocks the inhibition of the catalytic core exerted by the autoinhibitory sequence in the N-terminus, allowing the phosphorylation of substrate proteins [37, 75].

Although cell death in retinal degeneration is often believed to be driven by apoptosis, several studies hint at the involvement of a non-apoptotic cGMP/PKG-dependent cell death mechanism triggered by accumulation of cGMP in the degenerating photoreceptors [3, 77]. In situ hybridisation studies indicate that photoreceptors express the *Prkg1* gene [25], yet it is not clear whether this will lead to the expression of the PKG1 α or β isoform. Whether PKG2 is expressed in photoreceptors is not currently known. In animals showing elevated photoreceptor cGMP due to CNGC loss-of-function, PKG inhibition was found to be neuroprotective [54]. In *Cngb1*^−/−^ x *Prkg1*^−/−^ double mutant mice, in which both CNGC and PKG are dysfunctional, photoreceptor degeneration was markedly delayed when compared to *Cngb1*^−/−^ single mutants [108].

Taken together, PKG activation by high levels of photoreceptor cGMP is very likely to play an important role in triggering photoreceptor cell death. As far as the mechanisms triggered by high cGMP and PKG are concerned, two important questions remain: (1) At present it is not entirely clear which PKG isoform is responsible for photoreceptor cell death, a question that may be further addressed with conditional PKG knockout studies. (2) As a kinase, PKG has at least several hundred potential phosphorylation targets. While for instance the ryanodine receptor-2 [113] or CNGC modulation may in part mediate PKG-dependent effects [12], phosphoproteomic studies might help to identify which of these are most relevant for photoreceptor cell death and could perhaps highlight further targets for future therapy development.

### PDEs: where cGMP, cAMP, and Ca^2+^ signalling pathways (may) meet

To terminate cGMP-signalling, the cGMP molecule is broken down by enzymes belonging to the phosphodiesterase (PDE) group, which specifically hydrolyse the 3′5′ cyclic phosphate bond. Interestingly, there is a potential for crosstalk between cGMP-, cyclic Adenosine-Mono-Phosphate (cAMP)-, and Ca^2+^ signalling via PDEs. There are 11 structurally related but functionally distinct mammalian PDE gene families (PDEs 1–11). PDEs 5, 6, and 9 are specific for cGMP; PDEs 4, 7, and 8 hydrolyse cAMP only; while PDEs 1, 2, 3, 10, and 11 can degrade both cGMP and cAMP [15]. Among all the PDEs, the photoreceptor specific PDE6 has the highest cGMP turn-over rates [32, 51]. To date, a clear association with human disease is only known for mutations in genes coding for PDE6, as for example, in certain cases of RP and achromatopsia.

One possible connection between cyclic nucleotide signalling and Ca^2+^ is via adenylyl cyclase (AC), which is stimulated by Ca^2+^ to produce cAMP [106]. cAMP-signalling may be relevant for photoreceptor physiology, for instance, for the regulation of phototransduction sensitivity in rods [4] and cGMP stimulated CNGC-mediated Ca^2+^ influx could conceivably raise photoreceptor cAMP levels. Moreover, cGMP activates the cAMP-specific PDE2, while it inhibits cAMP-specific PDE3. Thus, depending on the PDE expression pattern in a given cell type, cGMP can either increase or decrease cAMP-signalling [15]. With regards to photoreceptor degeneration, an elevation of retinal cAMP was found in *rd2* mouse retina; however, it is unclear whether this increase was specific to photoreceptors or to other cell types of the retina [86]. In the *rd1* mouse, elevation of retinal cAMP content was shown to be restricted to the inner retina [53]. Since neither PDE2 nor PDE3 expression has been unambiguously demonstrated in photoreceptors, it is not currently known whether direct cGMP to cAMP crosstalk can occur in these cells.

## Ca^2+^ and calpain-type proteases

### Calpains: Ca^2+^ activated proteases and their role in photoreceptor cell death

Calpains are a group of Ca^2+^-dependent cysteine proteases. Even though their discovery dates back to 1964 [35], earlier than caspases or proteasomes, they remain enigmatic. The calpain family comprises 14 known isoforms [31]. The most ubiquitous and best characterized are calpain-1 and calpain-2. Also called µ-calpain and m-calpain, they are activated in vitro by micromolar and millimolar concentrations of Ca^2+^, respectively [31]. Since the physiological intracellular Ca^2+^ concentrations are thought to reach micromolar levels at most, calpain-1 is considered to be the most active isoform in vivo. Curiously, calpain-1 deficient mice present an apparently normal phenotype [5], while calpain-2 deficiency is embryonic lethal [19, 95], suggesting that calpain-2 has more important functions than calpain-1 in vivo.

Calpain-1 and calpain-2 appear to have opposing functions in the brain [7]. While calpain-1 activation has been linked to synaptic plasticity and neuroprotection, calpain-2 activity limits the extent of plasticity and contributes to neurodegeneration. CNGC-mediated excessive Ca^2+^-influx due to accumulation of cGMP may contribute to calpain protease activity, which was found to be associated to photoreceptor cell death [49, 50, 72]. Accordingly, calpain activity is increased in the outer nuclear layer in several models for retinal degeneration [3, 67]. Most of this increase in calpain activity is likely caused by calpain-2, with additional contributions from calpain-1 [78]. Remarkably, calpain-2 is activated by millimolar Ca^2+^ concentrations, thus suggesting that when calpain-2 activation occurs, the cell may have entered a degenerative stage at which it can no longer maintain the normal intracellular Ca^2+^ homeostasis (approx. 500 nm). Thus, calpain-2 activation may happen relatively late during the final stages of cell death.

The inhibition of calpains with SNJ-1945 decreased cell death in a rat model of photoreceptor degeneration induced by *N*-methyl-*N*-nitrosourea (MNU) [62], while the calpain inhibitors ALLN and ALLM reduced photoreceptor degeneration in the *rd1* mouse model for RD [85]. The treatment with calpastatin, considered to be the most specific inhibitor of calpains and inhibiting calpain isoforms 1, 2, and 9 (Suzuki et al. 2004), reduced calpain activity and cell death both in vitro and in vivo in the *rd1* mouse [72]. Overall, these studies indicate that Ca^2+^-activated calpain-type proteases are likely contributors to photoreceptor cell death.

### Downstream of calpains: AIF and PARP activation

The activity of calpain may contribute to retinal degeneration via activation of apoptosis-inducing factor (AIF) [85]. The AIF protein was first identified as an apoptogenic factor released from mitochondria to mediate caspase-independent apoptosis in mammals [94]. Nowadays, AIF is commonly associated with non-apoptotic cell death [91], which arguably is the most important degenerative mechanism in RD [3]. In retinal degeneration, the activation of AIF also decreases after blocking calpain activity [66]. Inhibition of calpain or chelation of Ca^2+^ prevents processing and release of AIF [61]. In the retinal ganglion cell 5 (RGC-5) line, calpain may induce necroptosis via AIF-modulation [91]. All the evidence indicates that AIF could also be one of the downstream targets of calpain, but it remains unclear how calpain may mediate AIF cleavage and contribute to non-apoptotic cell death.

Enzymes belonging to the 18-member poly(ADP-ribose) polymerase (PARP) family are involved in the repair of DNA damage but have also been linked to a variety of disease conditions [60, 89]. They consume NAD^+^ to add poly(ADP-ribose) polymers to target proteins in response to a variety of cellular stresses [13]. Overactivation of the most studied PARP isoform, PARP-1, is associated with elevations in intracellular Ca^2+^, indicating that both may take part in a common cell death pathway [18, 30, 117]. In RD, calpain and PARP have been proposed to independently contribute to cell death [3]. Yet, calpain may cleave many proteins that are vital for cell survival [70], including PARP-1 [56]. In addition, the activation of PARP-1 may require the activation of calpains [84]. Similarly, in the *N*-methyl-d-aspartic acid (NMDA) toxicity model in rat primary cortical neurons, PARP and calpain were found to be linked via PARP-1 induced alterations in mitochondrial Ca^2+^ homeostasis [104]. In a mouse model of controlled cortical impact (CCI), the PARP inhibitor PJ34 suppressed the over-activation of calpain [97]. Hence, in hereditary retinal degeneration, PARP may be linked directly to calpain activity or vice versa*.* One possible connection could be the excessive consumption of NAD^+^ by overactivated PARP-1. Since the intracellular pools of NAD^+^ and ATP are linked, the depletion of NAD^+^ could lead to a breakdown of photoreceptor energy metabolism (see below). Future studies may reveal the nature of relationship between calpains and PARP in retinal degeneration.

## cGMP and Ca^2+^: relation to photoreceptor energy metabolism

To maintain their OS dark current and to achieve single photon light sensitivity [36], photoreceptors consume disproportionately large amounts of energy [99]. Although retinal energy metabolism was studied already in the 1920s by Otto Warburg [109], even today surprisingly, little is known about how photoreceptors satisfy their enormous energy demand. Below and in Fig. 2, we briefly illustrate how cGMP and Ca^2+^ may influence photoreceptor metabolism and energy consumption.

The highest energy demanding function in a photoreceptor is the active transport of ions against their concentration and electrical gradients [111]. As mentioned above, the dark current is driven by the Na^+^ and K^+^ ion gradient. The Na^+^ gradient is maintained by the activity of the ATP-driven NKX in the IS [110]. Remarkably, NKX activity alone is responsible for the consumption of at least 50% of the ATP produced by photoreceptors [2], and cones appear to consume twice as much energy as rods [39]. On the other hand, in most mammalian retinas, rods outnumber cones and rod energy consumption is at least 75% lower in light, so that, a duplex retina provides for very high light sensitivity at an overall relatively low energy expenditure, when compared, for instance, with rhabdomeric insect photoreceptors [63]. This, however, no longer applies under conditions of an abnormal increase of cGMP concentration. Here, CNGCs are continuously activated, the influx of Na^+^ and Ca^2+^ increases further, and the photoreceptor membrane potential becomes more positive than it would otherwise be in darkness. This requires extra NKX activity and likely puts additional strain on photoreceptor energy metabolism.

Moreover, the continuous depolarization triggers a sustained activation of voltage-gated Ca^2+^ channels (VGCC) in the IS, cell body, and synapse [40, 105]. In these cellular compartments, the Ca^2+^ influx is counterbalanced by the ATP-driven plasma membrane Ca^2+^-ATPase (PMCA) [14]. Hence, via the intermediary of VGCC the overactivation of CNGC by high levels of OS cGMP may increase ATP consumption also in all other compartments of the photoreceptor cell. In summary, cGMP and Ca^2+^-signalling impact photoreceptor energy metabolism in ways that are likely to have a bearing for retinal disease pathogenesis.

## cGMP and Ca^2+^ signalling: opportunities for therapy development

### Strategies targeting Ca^2+^-influx

Paradoxically, photoreceptor degeneration has been reported to be linked to both increased and decreased intracellular Ca^2+^ levels, alternative hypotheses that may be referred to as “high Ca^2+^” and “low Ca^2+^” hypotheses, respectively [21, 26]. Thus, specific components of Ca^2+^ signalling might be targeted to delay RD progression. Pharmacological inhibition of Ca^2+^ channels has been suggested as a means to slow down degeneration in *rd1* [28, 96, 102]. Knockout studies on the *Cngb1*^−/−^ x *rd1* [68] and *Cngb1*^−/−^ x *rd10* [107] double-mutants suggested an important role for CNGC in retinal degeneration. On the other hand, similar studies on VGCC indicated that these Ca^2+^ permeable channels might be of lesser importance for photoreceptor cell death [82, 88]. Because of the multiple roles of Ca^2+^ signalling in cellular physiology, any targeting of Ca^2+^ permeable channels must be very specific. In case of RP, only rod-specific channels should be inhibited to avoid interference with cone-mediated vision. While specific pharmacological inhibition of channels may be feasible in some cases [16], genetic approaches using, for instance, antisense oligonucleotides [29] may allow for more selective therapeutic intervention. On the other hand, low levels of intracellular Ca^2+^ may also be detrimental to photoreceptors [21], raising doubts as to whether Ca^2+^ permeable channels really are a suitable target for pharmacological inhibition intervention.

### Strategies targeting cGMP-signalling

Since the imbalance of cGMP levels seems to be one of the initial events in photoreceptor cell death, neuroprotective strategies targeting upstream events may prevent or slow down the course of the disease. One way to target cGMP-signalling is to reduce the intracellular pool of guanine nucleotides [24]. For instance, inhibition of inosine monophosphate dehydrogenase (IMPDH) with mycophenolate mofetil (MMF), a prodrug of mycophenolic acid, suppresses de novo guanine nucleotide production [1]. Early treatment with MMF has been demonstrated to exert neuroprotection in *rd1* and *rd10* murine models, reducing photoreceptor cGMP levels and inhibiting cGMP-dependent cytotoxicity [114].

Effectors downstream of the cGMP-signalling cascade, such as CNGCs or PKG, may also be considered possible targets [100]. In particular, the idea of PKG as a potential target for neuroprotective strategies has been pointed out by different studies [3, 69, 108]. There are only a few PKG-specific inhibitors available so far. The oligopeptide DT-2 is a substrate-binding site inhibitor, highly specific for purified PKG Iα/β enzymes, even though DT2 was found to be inefficient in different cell types [9]. Another PKG inhibitor is KT5823, an ATP-binding site inhibitor. Despite its efficacy in vitro, KT5823 is also an inhibitor of PKA and PKC, making it ineffective in intact human platelets and rat mesangial cells [9]. A more specific inhibitor is a derivative of the fungal metabolite balanol, N46, which reduced thermal hyperalgesia and osteoarthritic pain in rats through selective inhibition of PKG Iα [93]. Finally, cGMP analogues constitute another class of PKG inhibitors that bind to the cGMP-binding domains of PKGs, without inducing the conformational changes needed for the activation of the kinase, leading to a competitive and reversible inhibition [98]. This class of PKG inhibitors showed marked neuroprotective properties in *rd1*, *rd2*, and *rd10* mice in vivo [103] suggesting that cGMP analogues may constitute a valid alternative for the study of PKG cellular functions, as well as for interventions in cGMP/PKG-dependent cell death mechanisms.

### The problem of retinal drug delivery

Delivery of therapeutic compounds or antisense oligonucleotides to photoreceptors remains a complicated task primarily due to the protected environment that these cells require. Drugs delivered by systemic administration methods need to cross either the inner blood-retinal barrier (iBRB) or outer blood-retinal barrier (oBRB) [52]. The iBRB consists of Müller cells surrounding the blood vessels of the inner retinal vasculature with endothelial cells connected via tight junctions. The oBRB is based on the RPE cells, which are also bound by tight junctions, and Bruch’s membrane, separating the retina from the dense vasculature in the choroid. To cross the BRB, glutathione-conjugated liposomes have been developed for active targeting of glutathione transporters [55, 103].

Local administration methods, such as intravitreal (IVT) injection, could circumvent parts of the BRB to make drug delivery more effective. Yet, rapid clearance of drug from the vitreous environment and the inner limiting membrane (ILM) at the vitreoretinal interphase still limits drug uptake by photoreceptors. To address the first issue, injectable drug-loaded implants have been developed based on hydrogel, providing sustained drug release for up to 6 months [116]. Non-biodegradable implants, though, can offer more delayed release for 2–3 years [41]. IVT administered micro- and nano- particle-based formulations have also been investigated. Among the microparticles, poly(l-lactide)-based particles have proved promising for retinal drug delivery. One system provided retention of small hydrophilic drugs in the retina for up to 3 months after IVT injection, while the drug solution administered without the delivery system could not be detected after 1 month. [92]. Unfortunately, micro-sized particles typically interfere with light transmission through the vitreous, which is why nanoparticles could be more suitable for IVT delivery to the retina. Particle diffusion in the vitreous depends largely on size and surface charge. The human vitreous has an average pore-size of 500–1000 nm in the center of the eye, and with the high concentration of hyaluronic acid (140–340 µg/mL), the vitreous is an overall anionic environment [90]. Hence, negatively charged nanoparticles generally diffuse faster in the vitreous than positively charged particles [20], which can aggregate [73]. However, positively charged nanoparticles grafted with shielding poly(ethylene glycol) (PEG) polymers have reached the retina following intravitreal injection [47]. While the pores in the ILM are too narrow for most nanoparticles to freely diffuse through, human serum albumin–based nanoparticles [44] and PEG-coated liposomes [11] have been shown to be taken up by Müller glial cells at the ILM and subsequently reached the deeper retinal tissue. In the future, successful therapy of retinal diseases will likely require the parallel development of therapeutic compounds together with a suitable drug delivery system that can overcome the relevant barriers.

## Conclusion

In the past 25 years, attempts at treatment development for retinal degeneration mirrored those pursued for neurodegenerative brain diseases. These attempts were based on two main premises, namely that (1) photoreceptor degeneration was governed by apoptosis as the causative cell death mechanism [26, 76], and (2) that apoptosis was triggered by high intracellular Ca^2+^ levels [65]. Accordingly, development efforts focused on lowering photoreceptor intracellular Ca^2+^ levels, often by targeting Ca^2+^-permeable channels [28, 102]. Unfortunately, these efforts failed to deliver a viable therapeutic option for RD (reviewed in [6]), suggesting that Ca^2+^ was in fact not as important as thought. In addition, in recent years, it has become increasingly clear that apoptosis—while involved in retinal development—is unlikely to be related to cell death caused by RD-gene mutations [3, 77].

In many genetically distinct types of RD, the rise in intracellular cGMP can be directly linked to photoreceptor degeneration [71, 77]. While cGMP in photoreceptors can target PKG and CNGC (Fig. 2), the deleterious effect of high cGMP likely stems from PKG activity, which was found to be both necessary and sufficient to cause cGMP-dependent photoreceptor death [69]. Further evidence comes from comparing PDE6 gene mutations (high cGMP and high Ca^2+^) with CNGC mutations (high cGMP but low Ca^2+^). While Ca^2+^ levels are elevated in only one of the two situations, cGMP is elevated in both, strongly suggesting that photoreceptor cell death was driven by cGMP-dependent processes. Nevertheless, the added strain on energy metabolism that may be induced by increased Ca^2+^ influx may aggravate the situation further, so that, when high cGMP and high Ca^2+^ come together, cell death is precipitated. Therefore, in certain genetic constellations targeting Ca^2+^-signalling may be considered for adjuvant treatment, to maximize the effectiveness of cGMP-targeting approaches.

In summary, the evidence available today suggests that therapeutic approaches in RD should focus on targeting cGMP-signalling rather than Ca^2+^-signalling. Indeed, recent studies have shown that interference with cGMP signalling, when combined with a suitable drug delivery vehicle, holds great promise for RD treatment development. Future pre-clinical studies shall attempt to validate these findings and hopefully subsequent clinical trials will translate them into mutation-independent therapy.

## References

[1] Allison AC, Kowalski WJ, Muller CD, Eugui EM (1993). Mechanisms of action of mycophenolic acid. Ann N Y Acad Sci.

[2] Ames A (1992). Energy requirements of CNS cells as related to their function and to their vulnerability to ischemia: a commentary based on studies on retina. Can J Physiol Pharmacol.

[3] Arango-Gonzalez B, Trifunović D, Sahaboglu A, Kranz K, Michalakis S, Farinelli P, Koch S, Koch F, Cottet S, Janssen-Bienhold U, Dedek K, Biel M, Zrenner E, Euler T, Ekström P, Ueffing M, Paquet-Durand F (2014). Identification of a common non-apoptotic cell death mechanism in hereditary retinal degeneration. PLoS ONE.

[4] Astakhova LA, Samoiliuk EV, Govardovskii VI, Firsov ML (2012). cAMP controls rod photoreceptor sensitivity via multiple targets in the phototransduction cascade. J Gen Physiol.

[5] Azam M, Andrabi SS, Sahr KE, Kamath L, Kuliopulos A, Chishti AH (2001). Disruption of the mouse mu-calpain gene reveals an essential role in platelet function. Mol Cell Biol.

[6] Barabas P, Cutler PC, Krizaj D (2010). Do calcium channel blockers rescue dying photoreceptors in the Pde6b (rd1) mouse?. Adv Exp Med Biol.

[7] Baudry M (2019). Calpain-1 and calpain-2 in the brain: Dr. Jekill and Mr Hyde?. Curr Neuropharmacol.

[8] Bertelsen M, Jensen H, Bregnhoj JF, Rosenberg T (2014). Prevalence of generalized retinal dystrophy in Denmark. Ophthalmic Epidemiol.

[9] Burkhardt M, Glazova M, Gambaryan S, Vollkommer T, Butt E, Bader B, Heermeier K, Lincoln TM, Walter U, Palmetshofer A (2000). KT5823 inhibits cGMP-dependent protein kinase activity in vitro but not in intact human platelets and rat mesangial cells. J Biol Chem.

[10] Burns ME, Mendez A, Chen J, Baylor DA (2002). Dynamics of cyclic GMP synthesis in retinal rods. Neuron.

[11] Camelo S, Lajavardi L, Bochot A, Goldenberg B, Naud MC, Fattal E, Behar-Cohen F, de Kozak Y (2007). Ocular and systemic bio-distribution of rhodamine-conjugated liposomes loaded with VIP injected into the vitreous of Lewis rats. Molecular Vis.

[12] Castro LR, Schittl J, Fischmeister R (2010). Feedback control through cGMP-dependent protein kinase contributes to differential regulation and compartmentation of cGMP in rat cardiac myocytes. Circ Res.

[13] Chaitanya GV, Alexander JS, Babu PPJCC, Signaling (2010). PARP-1 cleavage fragments: signatures of cell-death proteases in neurodegeneration. Cell Commun Signal.

[14] Comitato A, Subramanian P, Turchiano G, Montanari M, Becerra SP, Marigo V (2018). Pigment epithelium-derived factor hinders photoreceptor cell death by reducing intracellular calcium in the degenerating retina. Cell Death Dis.

[15] Conti M, Beavo J (2007). Biochemistry and physiology of cyclic nucleotide phosphodiesterases: essential components in cyclic nucleotide signaling. Annu Rev Biochem.

[16] Das S, Popp V, Power M, Groeneveld K, Melle C, Rogerson L, Achury M, Schwede F, Strasser T, Euler T, Paquet-Durand F, Nache V (2020) Redefining the role of Ca2+-permeable channels in hereditary photoreceptor degeneration using the D- and L-cis enantiomers of diltiazem. BioRxiv. 2020.12.04.411827

[17] Dell’Orco D, Schmidt H, Mariani S, Fanelli F (2009). Network-level analysis of light adaptation in rod cells under normal and altered conditions. Mol Biosyst.

[18] Duan Y, Gross RA, Sheu SS (2007). Ca2+-dependent generation of mitochondrial reactive oxygen species serves as a signal for poly(ADP-ribose) polymerase-1 activation during glutamate excitotoxicity. J Physiol.

[19] Dutt P, Croall DE, Arthur JS, Veyra TD, Williams K, Elce JS, Greer PA (2006). m-Calpain is required for preimplantation embryonic development in mice. BMC Dev Biol.

[20] Eriksena AZ, Brewer J, Andresena TL, Urquharta AJ (2017). The diffusion dynamics of PEGylated liposomes in the intact vitreous of the ex vivo porcine eye: a fluorescence correlation spectroscopy and biodistribution study. Int J Pharm.

[21] Fain GL, Lisman JE (1999). Light, Ca2+, and photoreceptor death: new evidence for the equivalent-light hypothesis from arrestin knockout mice. Invest Ophthalmol Vis Sci.

[22] Fain GL, Matthews HR, Cornwall MC, Koutalos Y (2001). Adaptation in vertebrate photoreceptors. Physiol Rev.

[23] Farber DB, Lolley RN (1974). Cyclic guanosine monophosphate: elevation in degenerating photoreceptor cells of the C3H mouse retina. Science.

[24] Farber DB, Park S, Yamashita C (1988). Cyclic GMP-phosphodiesterase of rd retina: biosynthesis and content. Exp Eye Res.

[25] Feil S, Zimmermann P, Knorn A, Brummer S, Schlossmann J, Hofmann F, Feil R (2005). Distribution of cGMP-dependent protein kinase type I and its isoforms in the mouse brain and retina. Neuroscience.

[26] Fox DA, Poblenz AT, He L (1999). Calcium overload triggers rod photoreceptor apoptotic cell death in chemical-induced and inherited retinal degenerations. Ann N Y Acad Sci.

[27] Francis SH, Busch JL, Corbin JD, Sibley D (2010). cGMP-dependent protein kinases and cGMP phosphodiesterases in nitric oxide and cGMP action. Pharmacol Rev.

[28] Frasson M, Sahel JA, Fabre M, Simonutti M, Dreyfus H, Picaud S (1999). Retinitis pigmentosa: rod photoreceptor rescue by a calcium-channel blocker in the rd mouse. Nat Med.

[29] Garanto A, Chung DC, Duijkers L, Corral-Serrano JC, Messchaert M, Xiao R, Bennett J, Vandenberghe LH, Collin RW (2016). In vitro and in vivo rescue of aberrant splicing in CEP290-associated LCA by antisense oligonucleotide delivery. Hum Mol Genet.

[30] Geistrikh I, Visochek L, Klein R, Miller L, Mittelman L, Shainberg A, Cohen-Armon M (2011). Ca2+-induced PARP-1 activation and ANF expression are coupled events in cardiomyocytes. Biochem J.

[31] Goll DE, Thompson VF, Li H, Wei W, Cong J (2003). The calpain system. Physiol Rev.

[32] Granovsky AE, Artemyev NO (2001). A conformational switch in the inhibitory gamma-subunit of PDE6 upon enzyme activation by transducin. Biochemistry.

[33] Gray-Keller MP, Detwiler PB (1994). The calcium feedback signal in the phototransduction cascade of vertebrate rods. Neuron.

[34] Gross OP, Pugh EN, Burns ME (2012). Spatiotemporal cGMP dynamics in living mouse rods. Biophys J.

[35] Guroff G (1964). A neutral, calcium-activated proteinase from the soluble fraction of rat brain. J Biol Chem.

[36] Hagins WA, Penn RD, Yoshikami S (1970). Dark current and photocurrent in retinal rods. Biophys J.

[37] Hofmann F, Bernhard D, Lukowski R, Weinmeister P (2009) cGMP regulated protein kinases (cGK). Handb Exp Pharmacol 137-162. 10.1007/978-3-540-68964-5_8 10.1007/978-3-540-68964-5_8 19089329

[38] Huttl S, Michalakis S, Seeliger M, Luo DG, Acar N, Geiger H, Hudl K, Mader R, Haverkamp S, Moser M, Pfeifer A, Gerstner A, Yau KW, Biel M (2005). Impaired channel targeting and retinal degeneration in mice lacking the cyclic nucleotide-gated channel subunit CNGB1. J Neurosci.

[39] Ingram NT, Fain GL, Sampath AP (2020). Elevated energy requirement of cone photoreceptors. Proc Natl Acad Sci U S A.

[40] Ingram NT, Sampath AP, Fain GL (2020). Membrane conductances of mouse cone photoreceptors. J Gen Physiol.

[41] Joseph RR, Venkatraman SS (2017). Drug delivery to the eye: what benefits do nanocarriers offer?. Nanomedicine.

[42] Kaupp UB, Niidome T, Tanabe T, Terada S, Bonigk W, Stuhmer W, Cook NJ, Kangawa K, Matsuo H, Hirose T, Miyata T, Numa S (1989). Primary structure and functional expression from complementary DNA of the rod photoreceptor cyclic GMP-gated channel. Nature.

[43] Kennan A, Aherne A, Humphries P (2005). Light in retinitis pigmentosa. Trends Genet.

[44] Kim H, Robinson SB, Csaky KG (2009). Investigating the movement of intravitreal human serum albumin nanoparticles in the vitreous and retina. Pharm Res.

[45] Koch KW (1991). Purification and identification of photoreceptor guanylate cyclase. J Biol Chem.

[46] Kolb H (2003). How the retina works. Am Sci.

[47] Koo H, Moon H, Han H, Naa JH, Huh MS, Park JH, Woo SJ, Park KH, Chan Kwon I, Kim K, Kim H (2012). The movement of self-assembled amphiphilic polymeric nanoparticles in the vitreous and retina after intravitreal injection. Biomaterials.

[48] Korenbrot JI, Rebrik TI (2002). Tuning outer segment Ca2+ homeostasis to phototransduction in rods and cones. Adv Exp Med Biol.

[49] Kulkarni M, Trifunović D, Schubert T, Euler T, Paquet-Durand F (2016). Calcium dynamics change in degenerating cone photoreceptors. Hum Mol Genet.

[50] Kutluer M, Huang L, Marigo V (2020). Targeting molecular pathways for the treatment of inherited retinal degeneration. Neural Regen Res.

[51] Leskov IB, Klenchin VA, Handy JW, Whitlock GG, Govardovskii VI, Bownds MD, Lamb TD, Pugh EN, Arshavsky VY (2000). The gain of rod phototransduction: reconciliation of biochemical and electrophysiological measurements. Neuron.

[52] Liu L, Liu X (2019) Roles of drug transporters in blood-retinal barrier. In: Liu X, Pan G (eds) Drug transporters in drug disposition, effects and toxicity. Singapore: Springer Singapore, 467–504. 10.1007/978-981-13-7647-4_10

[53] Lolley RN, Schmidt SY, Farber DB (1974). Alterations in cyclic AMP metabolism associated with photoreceptor cell degeneration in the C3H mouse. J Neurochem.

[54] Ma H, Butler MR, Thapa A, Belcher J, Yang F, Baehr W, Biel M, Michalakis S, Ding XQ (2015). cGMP/protein kinase G signaling suppresses inositol 1,4,5-trisphosphate receptor phosphorylation and promotes endoplasmic reticulum stress in photoreceptors of cyclic nucleotide-gated channel-deficient mice. J Biol Chem.

[55] Maussang D, Rip J, Kregten J, Avd H, Svd P, Boom Bd, Reijerkerk A, Chen L, Boer Md, Gaillard P, Vries H (2016). Glutathione conjugation dose-dependently increases brain-specific liposomal drug delivery in vitro and in vivo. Drug Discov Today Technol.

[56] McGinnis KM, Gnegy ME, Park YH, Mukerjee N, Wang KKJB, Communications br (1999). Procaspase-3 and poly (ADP) ribose polymerase (PARP) are calpain substrates. Biochem Biophys Res Commun.

[57] McLaughlin ME, Sandberg MA, Berson EL, Dryja TP (1993). Recessive mutations in the gene encoding the beta-subunit of rod phosphodiesterase in patients with retinitis pigmentosa. Nat Genet.

[58] Michaelides M, Hunt DM, Moore AT (2004). The cone dysfunction syndromes. Br J Ophthalmol.

[59] Nakatani K, Yau KW (1988). Calcium and light adaptation in retinal rods and cones. Nature.

[60] Nguewa PA, Fuertes MA, Valladares B, Alonso C, Perez JM (2005). Poly(ADP-ribose) polymerases: homology, structural domains and functions. Novel therapeutical applications. Prog Biophys Mol Biol.

[61] Norberg E, Gogvadze V, Ott M, Horn M, Uhlen P, Orrenius S, Zhivotovsky B (2008). An increase in intracellular Ca2+ is required for the activation of mitochondrial calpain to release AIF during cell death. Cell Death Differ.

[62] Oka T, Nakajima T, Tamada Y, Shearer TR, Azuma M (2007). Contribution of calpains to photoreceptor cell death in N-methyl-N-nitrosourea-treated rats. Exp Neurol.

[63] Okawa H, Sampath AP, Laughlin SB, Fain GL (2008). ATP consumption by mammalian rod photoreceptors in darkness and in light. Curr Biol.

[64] Olshevskaya EV, Ermilov AN, Dizhoor AM (2002). Factors that affect regulation of cGMP synthesis in vertebrate photoreceptors and their genetic link to human retinal degeneration. Mol Cell Biochem.

[65] Orrenius S, Zhivotovsky B, Nicotera P (2003). Regulation of cell death: the calcium-apoptosis link. Nat Rev Mol Cell Biol.

[66] Ozaki T, Ishiguro S-i, Hirano S, Baba A, Yamashita T, Tomita H, Nakazawa MJPO (2013). Inhibitory peptide of mitochondrial μ-calpain protects against photoreceptor degeneration in rhodopsin transgenic S334ter and P23H rats. PLoS ONE.

[67] Paquet-Durand F, Azadi S, Hauck SM, Ueffing M, van Veen T, Ekstrom P (2006). Calpain is activated in degenerating photoreceptors in the rd1 mouse. J Neurochem.

[68] Paquet-Durand F, Beck S, Michalakis S, Goldmann T, Huber G, Muhlfriedel R, Trifunovic D, Fischer MD, Fahl E, Duetsch G, Becirovic E, Wolfrum U, van Veen T, Biel M, Tanimoto N, Seeliger MW (2011). A key role for cyclic nucleotide gated (CNG) channels in cGMP-related retinitis pigmentosa. Hum Mol Genet.

[69] Paquet-Durand F, Hauck SM, van Veen T, Ueffing M, Ekström P (2009). PKG activity causes photoreceptor cell death in two retinitis pigmentosa models. J Neurochem.

[70] Paquet-Durand F, Johnson L, Ekström P (2007). Calpain activity in retinal degeneration. J Neurosci Res.

[71] Paquet-Durand F, Marigo V, Ekstrom P (2019). RD genes associated with high photoreceptor cGMP-levels (mini-review). Adv Exp Med Biol.

[72] Paquet-Durand F, Sanges D, McCall J, Silva J, van Veen T, Marigo V, Ekström P (2010). Photoreceptor rescue and toxicity induced by different calpain inhibitors. J Neurochem.

[73] Peeters L, Sanders NN, Braeckmans K, Boussery K, Van de Voorde J, Smedt SCD, Demeester J (2005). Vitreous: a barrier to nonviral ocular gene therapy. Invest Ophthalmol Vis Sci.

[74] Peng C, Rich ED, Varnum MD (2004). Subunit configuration of heteromeric cone cyclic nucleotide-gated channels. Neuron.

[75] Piwkowska A, Rogacka D, Audzeyenka I, Kasztan M, Angielski S, Jankowski M (2016). Intracellular calcium signaling regulates glomerular filtration barrier permeability: the role of the PKGIα-dependent pathway. FEBS Lett.

[76] Portera-Cailliau C, Sung CH, Nathans J, Adler R (1994). Apoptotic photoreceptor cell death in mouse models of retinitis pigmentosa. Proc Natl Acad Sci U S A.

[77] Power M, Das S, Schutze K, Marigo V, Ekstrom P, Paquet-Durand F (2020). Cellular mechanisms of hereditary photoreceptor degeneration - focus on cGMP. Prog Retin Eye Res.

[78] Power MJ, Rogerson LE, Schubert T, Berens P, Euler T, Paquet-Durand F (2020). Systematic spatiotemporal mapping reveals divergent cell death pathways in three mouse models of hereditary retinal degeneration. J Comp Neurol.

[79] Pugh EN, Lamb TD (1990). Cyclic GMP and calcium: the internal messengers of excitation and adaptation in vertebrate photoreceptors. Vision Res.

[80] Pugh EN, Nikonov S, Lamb TD (1999). Molecular mechanisms of vertebrate photoreceptor light adaptation. Curr Opin Neurobiol.

[81] Ramamurthy V, Niemi GA, Reh TA, Hurley JB (2004). Leber congenital amaurosis linked to AIPL1: a mouse model reveals destabilization of cGMP phosphodiesterase. Proc Natl Acad Sci U S A.

[82] Read DS, McCall MA, Gregg RG (2002). Absence of voltage-dependent calcium channels delays photoreceptor degeneration in rd mice. Exp Eye Res.

[83] Reeves J, Condrescu M (2008). Ionic regulation of the cardiac sodium-calcium exchanger. Channels.

[84] Sacca E, Pizzutti N, Corazzin M, Lippe G, Piasentier E (2016). Assessment of calpain and caspase systems activities during ageing of two bovine muscles by degradation patterns of alphaII spectrin and PARP-1. Anim Sci J.

[85] Sanges D, Comitato A, Tammaro R, Marigo V (2006). Apoptosis in retinal degeneration involves cross-talk between apoptosis-inducing factor (AIF) and caspase-12 and is blocked by calpain inhibitors. Proc Natl Acad Sci U S A.

[86] Sanyal S, Fletcher R, Liu YP, Aguirre G, Chader G (1984). Cyclic nucleotide content and phosphodiesterase activity in the rds mouse (020/A) retina. Exp Eye Res.

[87] Schnetkamp PP (1995). How does the retinal rod Na-Ca+K exchanger regulate cytosolic free Ca2+?. J Biol Chem.

[88] Schon C, Paquet-Durand F, Michalakis S (2016). Cav1.4 L-type calcium channels contribute to calpain activation in degenerating photoreceptors of rd1 mice. PLoS ONE.

[89] Schreiber V, Dantzer F, Ame JC,  de Murcia G (2006). Poly(ADP-ribose): novel functions for an old molecule. Nat Rev Mol Cell Biol.

[90] Shafaiea S, Huttera V, Browna MB, Cooka MT, Chaua DYS (2018). Diffusion through the ex vivo vitreal body – bovine, porcine, and ovine models are poor surrogates for the human vitreous. Int J Pharm.

[91] Shang L, Huang J-F, Ding W, Chen S, Xue L-X, Ma R-F, Xiong K (2014). Calpain: a molecule to induce AIF-mediated necroptosis in RGC-5 following elevated hydrostatic pressure. BMC Neurosci.

[92] Shelke NB, Kadam R, Tyagi P, Rao VR, Kompella UB (2011). Intravitreal poly(L-lactide) microparticles sustain retinal and choroidal delivery of TG-0054, a hydrophilic drug intended for neovascular diseases. Drug Deliv Transl Res.

[93] Sung YJ, Sofoluke N, Nkamany M, Deng S, Xie Y, Greenwood J, Farid R, Landry DW, Ambron RT (2017). A novel inhibitor of active protein kinase G attenuates chronic inflammatory and osteoarthritic pain. Pain.

[94] Susin SA, Lorenzo HK, Zamzami N, Marzo I, Snow BE, Brothers GM, Mangion J, Jacotot E, Costantini P, Loeffler M, Larochette N, Goodlett DR, Aebersold R, Siderovski DP, Penninger JM, Kroemer G (1999). Molecular characterization of mitochondrial apoptosis-inducing factor. Nature.

[95] Takano J, Mihira N, Fujioka R, Hosoki E, Chishti AH, Saido TC (2011). Vital role of the calpain-calpastatin system for placental-integrity-dependent embryonic survival. Mol Cell Biol.

[96] Takano Y, Ohguro H, Dezawa M, Ishikawa H, Yamazaki H, Ohguro I, Mamiya K, Metoki T, Ishikawa F, Nakazawa M (2004). Study of drug effects of calcium channel blockers on retinal degeneration of rd mouse. Biochem Biophys Res Commun.

[97] Tao X, Chen X, Hou Z, Hao S, Liu B (2017). Protective functions of PJ34, a poly(ADP-ribose) polymerase inhibitor, are related to down-regulation of calpain and nuclear factor-κB in a mouse model of traumatic brain injury. World Neurosurg.

[98] Tolone A, Belhadj S, Rentsch A, Schwede F, Paquet-Durand F (2019) The cGMP pathway and inherited photoreceptor degeneration: targets, compounds, and biomarkers. Genes (Basel) 10. 10.3390/genes10060453 10.3390/genes10060453 PMC6627777 31207907

[99] Trick GL, Berkowitz BA (2005). Retinal oxygenation response and retinopathy. Prog Retin Eye Res.

[100] Trifunović D, Sahaboglu A, Kaur J, Mencl S, Zrenner E, Ueffing M, Arango-Gonzalez B, Paquet-Durand F (2012). Neuroprotective strategies for the treatment of inherited photoreceptor degeneration. Curr Mol Med.

[101] Tucker CL, Woodcock SC, Kelsell RE, Ramamurthy V, Hunt DM, Hurley JB (1999). Biochemical analysis of a dimerization domain mutation in RetGC-1 associated with dominant cone-rod dystrophy. Proc Natl Acad Sci U S A.

[102] Vallazza-Deschamps G, Cia D, Gong J, Jellali A, Duboc A, Forster V, Sahel JA, Tessier LH, Picaud S (2005). Excessive activation of cyclic nucleotide-gated channels contributes to neuronal degeneration of photoreceptors. Eur J Neurosci.

[103] Vighi E, Trifunovic D, Veiga-Crespo P, Rentsch A, Hoffmann D, Sahaboglu A, Strasser T, Kulkarni M, Bertolotti E, van den Heuvel A, Peters T, Reijerkerk A, Euler T, Ueffing M, Schwede F, Genieser HG, Gaillard P, Marigo V, Ekstrom P, Paquet-Durand F (2018). Combination of cGMP analogue and drug delivery system provides functional protection in hereditary retinal degeneration. Proc Natl Acad Sci U S A.

[104] Vosler PS, Sun D, Wang S, Gao Y, Kintner DB, Signore AP, Cao G, Chen J (2009). Calcium dysregulation induces apoptosis-inducing factor release: cross-talk between PARP-1- and calpain-signaling pathways. Exp Neurol.

[105] Waldner DM, Bech-Hansen NT, Stell WK (2018) Channeling vision: CaV1.4-a critical link in retinal signal transmission. Biomed Res Int 2018: 7272630. 10.1155/2018/7272630 10.1155/2018/7272630 PMC5966690 29854783

[106] Wang H, Zhang M (2012). The role of Ca(2)(+)-stimulated adenylyl cyclases in bidirectional synaptic plasticity and brain function. Rev Neurosci.

[107] Wang T, Reingruber J, Woodruff ML, Majumder A, Camarena A, Artemyev NO, Fain GL, Chen J (2018). The PDE6 mutation in the rd10 retinal degeneration mouse model causes protein mislocalization and instability and promotes cell death through increased ion influx. J Biol Chem.

[108] Wang T, Tsang SH, Chen J (2017). Two pathways of rod photoreceptor cell death induced by elevated cGMP. Hum Mol Genet.

[109] Warburg O (1925). The metabolism of carcinoma cells. J Cancer Res.

[110] Wetzel RK, Arystarkhova E, Sweadner KJ (1999). Cellular and subcellular specification of Na, K-ATPase alpha and beta isoforms in the postnatal development of mouse retina. J Neurosci.

[111] Wong-Riley MT (2010). Energy metabolism of the visual system. Eye Brain.

[112] Xu J, Morris L, Thapa A, Ma H, Michalakis S, Biel M, Baehr W, Peshenko IV, Dizhoor AM, Ding XQ (2013). cGMP accumulation causes photoreceptor degeneration in CNG channel deficiency: evidence of cGMP cytotoxicity independently of enhanced CNG channel function. J Neurosci.

[113] Yang F, Ma H, Butler MR, Ding XQ (2020). Potential contribution of ryanodine receptor 2 upregulation to cGMP/PKG signaling-induced cone degeneration in cyclic nucleotide-gated channel deficiency. FASEB J.

[114] Yang P, Lockard R, Titus H, Hiblar J, Weller K, Wafai D, Weleber RG, Duvoisin RM, Morgans CW, Pennesi ME (2020). Suppression of cGMP-dependent photoreceptor cytotoxicity with mycophenolate is neuroprotective in murine models of retinitis pigmentosa. Invest Ophthalmol Vis Sci.

[115] Yau KW (1994). Phototransduction mechanism in retinal rods and cones. The Friedenwald Lecture. Invest Ophthalmol Vis Sci.

[116] Yu Y, Lau LCM, Lo AC-y, Chau Y (2015). Injectable chemically crosslinked hydrogel for the controlled release of bevacizumab in vitreous: a 6-month in vivo study. Transl Vis Sci Technol.

[117] Zhang F, Xie R, Munoz FM, Lau SS, Monks TJ (2014). PARP-1 hyperactivation and reciprocal elevations in intracellular Ca2+ during ROS-induced nonapoptotic cell death. Toxicol Sci.

